# Endoscopic features of deficient mismatch repair/microsatellite instability‐high and BRAF‐mutated colorectal cancer

**DOI:** 10.1002/deo2.70132

**Published:** 2025-05-04

**Authors:** Rika Omote, Shizuma Omote, Ryohei Sumii, Joichiro Horii, Isao Fujita, Hideaki Miyaso, Tatsuya Toyokawa, Masaru Inagaki

**Affiliations:** ^1^ Department of Diagnostic Pathology NHO Fukuyama Medical Center Hiroshima Japan; ^2^ Department of Internal Medicine Fukuyama Minami Hospital Hiroshima Japan; ^3^ Department of Gastroenterology NHO Fukuyama Medical Center Hiroshima Japan; ^4^ Department of Gastroenterology Okayama Rosai Hospital Okayama Japan; ^5^ Department of Surgery NHO Fukuyama Medical Center Hiroshima Japan

**Keywords:** BRAF, coagulative necrosis, colorectal cancer, dMMR/MSI‐high, yellow slough

## Abstract

**Objective:**

Recent advancements in genome analyses, including the BRAF gene and mismatch repair (MMR) gene/microsatellite instability (MSI), have revealed the biological diversity of colorectal cancer (CRC). BRAF‐mutated CRC has a poor prognosis; however, cases exhibiting deficient MMR (dMMR)/MSI‐high (MSI‐H) and BRAF gene mutations have demonstrated significant prognostic improvement following recent immune checkpoint inhibitor therapy. Therefore, the diagnosis of these subtypes is important. This study aimed to identify the endoscopic features of dMMR/MSI‐high and BRAF‐mutated CRCs.

**Methods:**

A retrospective analysis was conducted on 292 CRC cases. Clinicopathological findings, focusing on dMMR/MSI‐H and BRAF‐mutated subtypes, were determined. Endoscopic images were analyzed for the presence of yellow slough. Surface material characteristics were assessed through a histopathological evaluation.

**Results:**

Of the 256 cases analyzed, 27 were dMMR/MSI‐H CRC, including 12 BRAF‐mutant cases. Yellow slough was observed in 83.3% of dMMR/MSI‐H and BRAF‐mutated CRCs, compared with 13.3% dMMR/MSI‐H and BRAF wild‐type CRCs and 1.3% pMMR/MSS and BRAF wild‐type CRCs. Histological examination showed a correlation of yellow slough with coagulative necrosis and thicker surface layers in dMMR/MSI‐high and BRAF‐mutated CRCs.

**Conclusion:**

Yellow slough on endoscopy may help identify dMMR/MSI‐H‐ and BRAF‐mutated CRC and allow the initiation of appropriate molecular testing and immunotherapy

## INTRODUCTION

Recent large‐scale genome analyses have revealed the molecular biological diversity of colorectal cancer (CRC). In the clinical setting, analyses of RAS and BRAF gene mutations to predict the efficacy of molecular target therapy, as well as analyses of mismatch repair (MMR) proteins, microsatellite instability (MSI) testing, and HER2 proteins, have become essential.^1^, ^2^, ^3^, ^4^ In particular, the development of immune checkpoint inhibitors for deficient MMR (dMMR)/MSI‐high (MSI‐H) CRC is progressing rapidly, and ipilimumab and nivolumab combination therapy are useful for chemotherapy in cases where surgical resection is not possible^5^ or as preoperative chemotherapy for resectable CRC.^6^ In contrast, BRAF gene mutations have been found in 5%–15% of CRC cases; are more common in older patients, women, and right‐sided colon cancers, and are characterized by a high frequency of advanced cancer, resistance to chemotherapy, and poor prognosis.^7^ In recent years, BRAF inhibitors have been proven effective in combination with MAPK/ERK kinase and epidermal growth factor receptor inhibitors in chemotherapy,^8^ and, similar to MMR and MSI, BRAF testing is becoming essential in considering treatment strategies; however, even with the aforementioned treatment, the prognosis remains poor. However, BRAF mutations are strongly associated with dMMR/MSI‐H (46%–75%),^9^, ^10^, ^11^ and some CRCs with BRAF mutations can benefit from remarkable improvements in prognosis achieved with recent immune checkpoint inhibitor development. Therefore, CRC with BRAF mutations and dMMR/MSI‐H should be clinically identified as a subtype in terms of treatment strategy. dMMR/MSI‐H‐ and BRAF‐mutated CRC are recognized as lesions in the serrated pathway that develop from sessile serrated lesions as early lesions.^12^, ^13^ Endoscopic findings of sessile serrated lesions with cancer include nodular elevations, depression, redness, nodular and granular changes, pedunculated or subpedunculated morphology, and interrupted blood vessels.^14^, ^15^, ^16^ However, the endoscopic features exhibited in d‐MMR/MSI‐H and BRAF‐mutated advanced cancer are unclear. This subtype of CRC has an extremely poor prognosis compared with existing treatments; however, it strongly benefits from immunotherapy; therefore, it is important to diagnose it early and link it to appropriate treatment. If the endoscopic features that clinicians can recognize are clear, early diagnosis is possible. In the course of analyzing the endoscopic images of dMMR/MSI‐H‐ and BRAF‐mutated CRCs at our hospital, we noticed that many cases showed yellow slough. Therefore, this study was conducted with the aim of clarifying the endoscopic features of dMMR/MSI‐H and BRAF‐mutated CRC.

## METHODS

We extracted clinical, endoscopic, and pathological findings from the medical records of 292 patients diagnosed with CRC at Fukuyama Medical Center between July 2021 and November 2024, who had undergone MMR protein or MSI testing and BRAF and RAS gene analysis. Regarding RAS/RAF testing, we used the polymerase chain reaction reverse sequence‐specific oligonucleotide method in all cases. Considering MSI/MMR testing, since October 2022, when the MMR protein test was approved to be under insurance coverage, we have been performing the MMR protein test and immunohistochemical staining method using four antibodies (MLH1, MSH2, PMS2, and MSH6; in 178 out of 292 cases). Cases before October 2022 (*n* = 114) underwent MSI tests using multiplex polymerase chain reaction and fragment analysis.

All procedures were performed using high‐definition endoscopic systems: the EVIS LUCERA ELITE CV‐290 series (Olympus Medical Systems) and the LASEREO series (Fujifilm Corporation). The specific endoscope models used included: PCF‐H290ZI, PCF‐Q260AZI, PCF‐PQ260I, PCF‐XP290N, and EC‐L600ZP.

**FIGURE 1:**
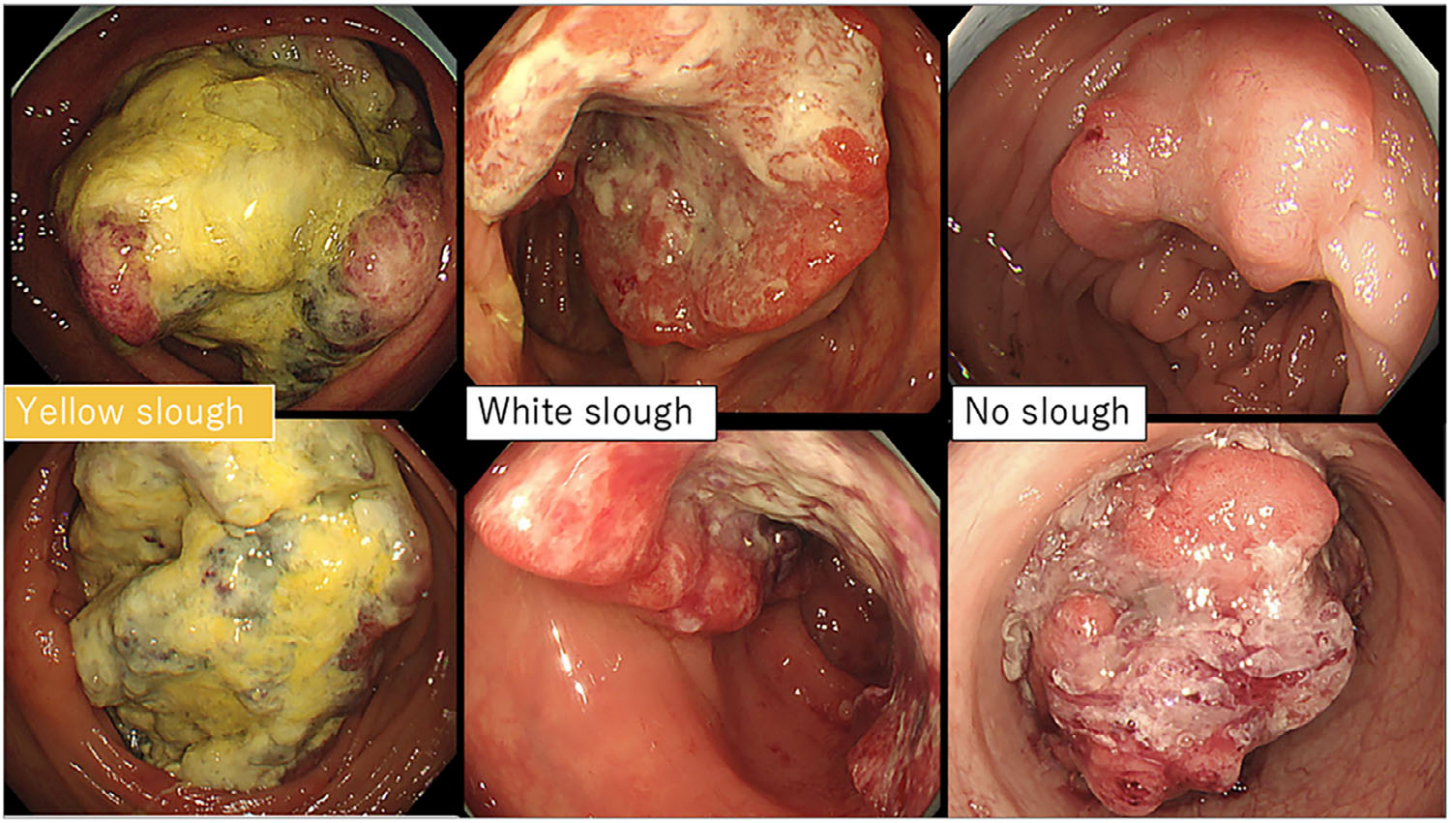
We classified cases into three categories: yellow slough (cases where a thick yellow adherent was observed on the surface of the colorectal tumor), white slough (cases where a white adherent was observed), and no slough (cases where no adherent or only mucus was observed).

Patients with appendiceal carcinoma, anal canal carcinoma, and anastomotic recurrence, which are difficult to assess endoscopically, and rare histological types, such as endocrine carcinoma and squamous cell carcinoma, were excluded. Furthermore, cases in which no preoperative endoscopy was performed, or in which endoscopic images were difficult to obtain because the examination was performed at another hospital, were excluded. Clinical staging was performed according to the 8th edition of the AJCC TNM system.^17^ Endoscopic images were analyzed by two gastroenterologists who assessed the superficial adherent material. Cases, wherein even one of the two gastroenterologists found it difficult to observe the tumor surface because of inadequate bowel cleansing or severe stenosis, were excluded. Only those cases in which surface adherents were sufficiently observable were selected. Yellow superficial adherent material was defined as yellow slough and white adherent material was defined as white slough. Two experts evaluated the endoscopic images after learning about two cases of yellow slough, two cases of white slough, and two cases with no adhesions or only mucus adhesions (Figure 1). Experts were blinded to the clinical information and assessed adherents using only endoscopic images. Yellow slough was concluded based on the agreement between the two experts. White slough was defined by one of the experts. One case in which only one expert recognized it as a yellow slough and the other recognized it as a white slough was finally considered a white slough.

Ulcer surface materials observed during colonoscopy were visually classified by two expert gastroenterologists into yellow (*n* = 15) or white (*n* = 49) groups. To validate this classification, regions of interest were extracted from endoscopic images.

Based on regions of interest in each endoscopic image, the mean color values were calculated and converted into the CIELAB (L*a*b*) color space using OpenCV (Python). Color differences (ΔE) and intergroup comparisons were subsequently analyzed.

Superficial adherents were histopathologically examined using surgical materials. Specimens with the deepest tumor invasion and the thickness and characteristics of the superficial adherents were examined. Cases of colorectal stent placement were excluded from this study to avoid affecting the adherence evaluation. Among the evaluated specimens, deposits with even the slightest signs of coagulative necrosis were judged as coagulative necrosis of the tumor. Statistical analyses were performed using SPSS Statistics version 25 for Windows (SPSS Inc.). The chi‐square test was used to analyze qualitative variables and the Mann‐Whitney U test was used to analyze quantitative data. Binary logistic regression analysis was used for multivariate analysis. *p* < 0.05 indicated statistical significance.

## RESULTS

**FIGURE 2:**
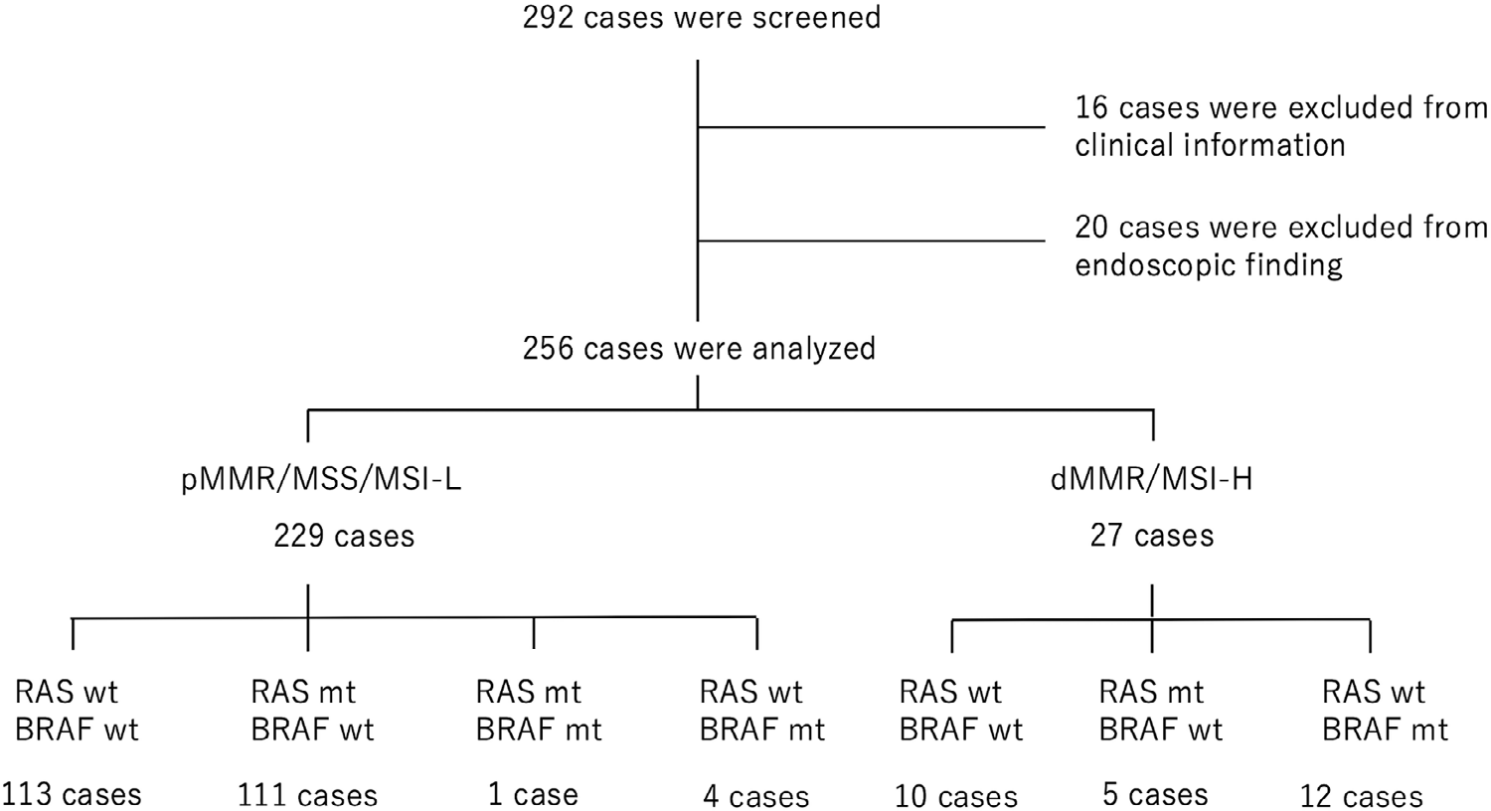
Of the 294 cases, 36 were excluded due to reasons, such as specific types of cancer, lack of endoscopic images, inappropriate prior treatment, or severe stenosis. A total of 256 cases were included in the analysis. These cases were classified according to the results of RAS, RAF, and mismatch repair/microsatellite instability (MMR/MSI) examinations.

Among the 294 cases, 16 were excluded, including nine cases of appendiceal cancer, one case of anal canal cancer, one case of anastomotic recurrence, three cases for which there were no endoscopic images, two cases of mixed neuroendocrine‐non‐neuroendocrine neoplasm, and one case of squamous cell carcinoma. Twenty cases were excluded because of poor preparation (four cases) or severe stenosis (16 cases) due to difficulties in endoscopic assessment. A total of 256 patients were analyzed (Figure 2). Of them, 229 had pMMR/MSS/MSI‐low CRCs and 27 had dMMR/MSI‐high CRCs. dMMR/MSI‐high CRCs accounted for 10.5% of all cases. pMMR/MSS/MSI‐low CRCs were divided into 113 cases of RAS wild type (RAS wt) and BRAF wild type (BRAF wt; RAS wt/BRAF wt), 111 cases of RAS mutated type (RAS mt) and BRAF wt (RAS mt/BRAF wt), four cases of RAS wt and BRAF mutated type (BRAFmt; RAS wt/BRAF mt), one case of RAS mt/BRAF mt. dMMR/MSI‐low CRCs were divided into 10 cases of RAS wt/BRAF wt, five cases of RAS mt/BRAF wt, and 12 cases of RAS wt/BRAF mt CRCs (Figure 2). The average age, sex, tumor location, macroscopic type, and stage are shown in Table 1. The average age of patients with pMMR/MSS/MSI‐low CRCs was almost the same, regardless of the presence or absence of RAS and BRAF gene mutations: RAS wt/BRAF wt group, 68.7 years; RAS mt/BRAF wt group, 69.0 years, and RAS wt/BRAF mt group, 69.6 years. Among dMMR/MSI‐high CRC cases, the average age was lesser, at 58 years for RAS wt/BRAF wt and 46.8 years for RAS mt/BRAF wt, while the average age was higher for the RAS wt/BRAF mt group at 74.5 years. In terms of gender, the proportion of female patients was 32.7% for RAS wt/BRAF wt, 58.6% for RAS mt/BRAF wt, and 50% for RAS wt/BRAF mt in pMMR/MSS/MSI‐low CRC, and 63.6% for RAS wt/BRAF wt, 20% for RAS mt/BRAF wt, and 66.7% for RAS wt/BRAF mt in dMMR/MSI‐high CRC cases.

**TABLE 1:** Analysis of 256 cases of colorectal cancer.

	pMMR/MSS/MSI‐low			dMMR/MSI‐high		
	RAS wt/BRAF wt	RAS mt/BRAF wt	RAS wt/BRAF mt	RAS wt/BRAF wt	RAS mt/BRA Fwt	RAS wt/BRAF mt
Case	113	111	4	10	5	12
Age (years)	68.7 (±22.0)	69.0 (±21.5)	69.6 (±21.5)	58 (±37.2)	46.8 (±39.9)	74.5 (±14.2)
Sex (female %)	37 (32.7%)	54 (48.6%)	2 (50%)	7 (63.6)	1 (20%)	8 (66.7%)
Location						
Right side	22	38	2	6	1	12
Left side	102	83	2	5	4	0
Macroscopic classification						
Type 1	6	20	0	1	1	1
Type 2	109	95	4	10	3	8
Type 3	1	1	0	0	0	1
Type 4	0	1	0	1	0	1
Stage						
I	9	5	0	1	0	0
II	35	33	2	10	3	2
III	46	35	2	0	1	7
IV	30	47	0	1	0	3

As there was only one case of RAS mutated type/RAF mutated type, it was not included in the table. We examined age, sex, location, macroscopic classification, and stage in the other 255 cases.

**FIGURE 3:**
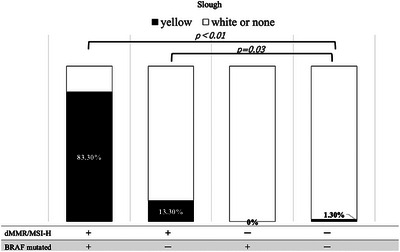
Yellow slough was observed in 83.3% of deficient mismatch repair/microsatellite instability‐high (dMMR/MSI‐high) and BRAF mt colorectal cancers. In contrast, it appeared in 13.3% of dMMR/MSI‐high and BRAF wt cases, 0% of pMMR/MSS/MSI‐low and BRAF mt cases, and 1.3% of pMMR/MSS/MSI‐low and BRAF wt cases.

**FIGURE 4:**
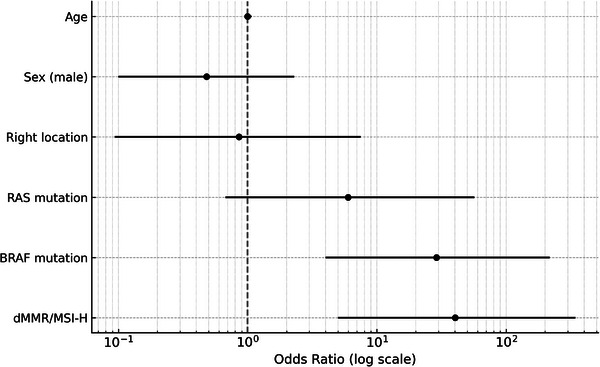
Multivariate analysis showed BRAF mutation and deficient mismatch repair/microsatellite instability‐high (dMMR/MSI‐H) as independent risk factors. 95% confidence intervals for odds ratios on the logarithmic scale.

**FIGURE 5:**
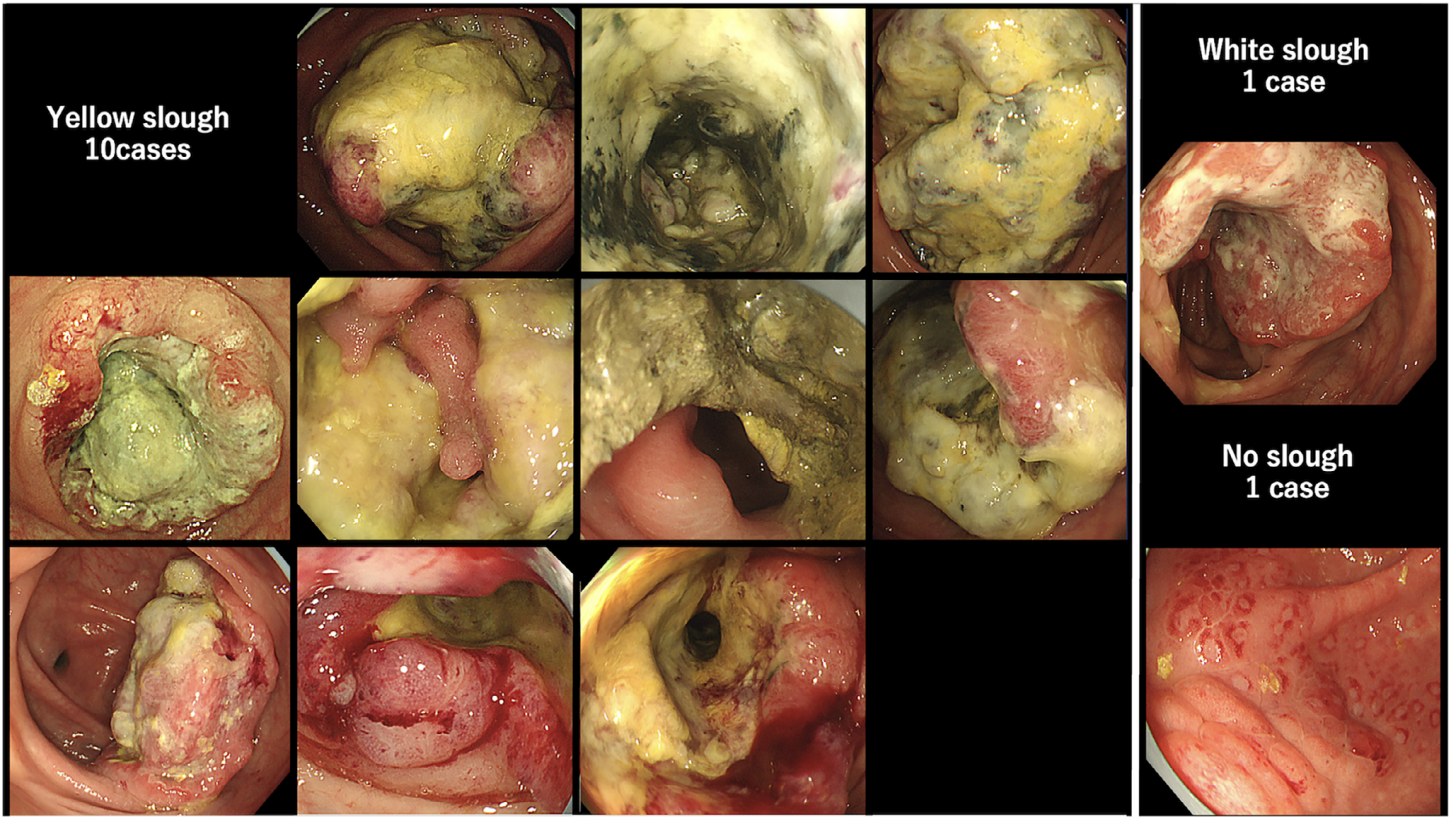
Endoscopic images of all 12 cases of deficient mismatch repair/microsatellite instability‐high and BRAF mt colorectal cancers. Ten of the 12 cases showed yellow slough. One case showed a white slough and the other showed a special endoscopic image similar to a submucosal tumor.

**FIGURE 6:**
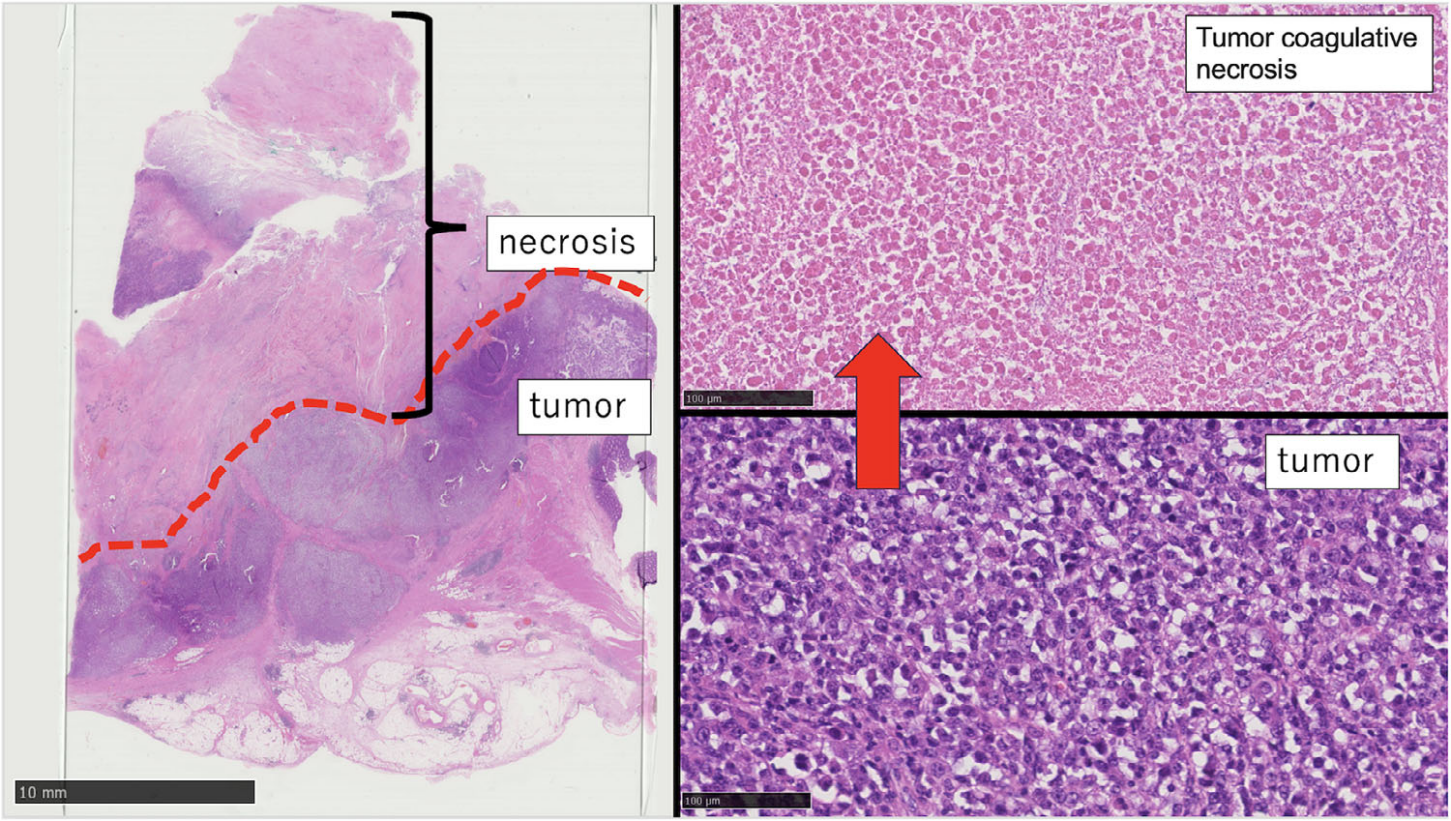
The surface adherent matter of the tumor was examined using the section with the deepest depth of invasion of the cancer in the surgical tissue. A thick necrotic layer was observed on the surface under low magnification, and under high magnification, the nuclei disappeared while maintaining the shape of the tumor. This was thought to be a coagulative necrosis image of the tumor.

Inter‐rater agreement between the two gastroenterologists who evaluated surface deposits (yellow slough, white slough, or no slough) was assessed using Cohen's κ coefficient. The κ value was 0.827 (*p *< 0.01), which, according to the Landis and Koch criteria, indicated almost perfect agreement. These results indicated a high degree of interobserver reliability in evaluating surface deposits. The mean CIELAB values (± standard deviation) in the yellow group were L*: 138.0 ± 21.8, a*: 148.7 ± 4.1, and b*: 159.6 ± 6.1, whereas those in the white group were L*: 177.3 ± 28.6, a*: 124.5 ± 12.8, and b*: 169.6 ± 10.2. All parameters showed significant differences (*p* < 0.001). The ΔE between the groups was 47.2, indicating a substantial perceptual difference and supporting the validity of the visual classification. The overall classification accuracy (accuracy) was 91.1%, indicating that distinguishing between the two groups with high accuracy was possible. Endoscopic findings showed that 10 of the 12 patients with dMMR/MSI‐high and BRAF mt CRCs had yellow slough (83.3%). In contrast, yellow slough was observed in two of 15 cases (13.3%) of dMMR/MSI‐high and BRAF wt CRCs, none of the four cases (0%) of pMMR/MSS/MSI‐low and BRAF mt CRCs, and in three of 227 cases (1.3%) of pMMR/MSS/MSI‐low and BRAF wt CRC cases (Table 2, Figure 3). Multivariate analysis revealed that BRAF mutations and dMMR/MSI‐H were independent risk factors (Figure 4). In the multivariate analysis, dMMR/MSI‐H positivity and BRAF mutation were independent risk factors for yellow slough (Figure 4). Although the number of yellow slough cases was relatively small (*n* = 15), we applied Firth's penalized likelihood logistic regression to reduce small‐sample bias. Furthermore, a post hoc power analysis using an estimated odds ratio of 30 yielded a power of over 99.9% (α = 0.05). Endoscopic images of all 12 cases of dMMR/MSI‐H and BRAF mt CRCs are shown in Figure 5. In these cases, two cases did not show yellow slough, one showed white slough, and the other showed a specific endoscopic image that extended submucosally. Subsequently, histological images of the surgical materials were examined for superficial adherence. Surface adhesions contained fibrin, necrotic material, granulation tissue, and inflammatory cell infiltration; in some cases, tumor coagulative necrosis was observed (Figure 6). Tumor coagulative necrosis was observed only in the cases of yellow slough cases, and all cases were dMMR/MSI‐high and BRAF mt CRCs. It was observed in six of seven cases (85.7%) of dMMR/MSI‐high and BRAF mt CRCs.

**TABLE 2:** Slough type by RAS/RAF and MMR subtypes in colorectal cancer.

	pMMR/MSS/MSI‐low			dMMR/MSI‐high		
	RASwt/BRAFwt	RASmt/BRAFwt	RASwt/BRAFmt	RASwt/BRAFwt	RASmt/BRAFwt	RASwt/BRAFmt
Yellow slough	0	3	0	1	1	10
White slough	21	22	2	1	2	1
No slough	92	86	2	8	2	1

In the 12 cases of deficient mismatch repair/microsatellite instability‐high and RASwt/BRAFmt colorectal cancer (CRC), 10 had yellow slough, while in the cases of pMMR/MSS/MSI‐low CRC, we observed white slough or no slough in many cases.

**FIGURE 7:**
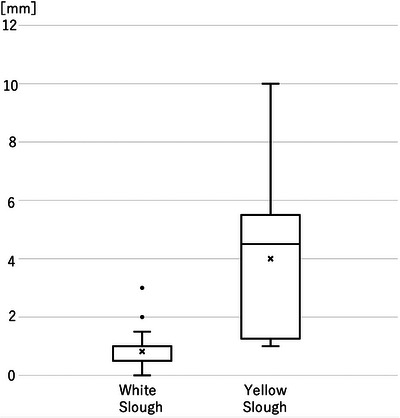
The thickness of the tissue adhesion of surgical materials was analyzed in nine cases of yellow and 20 cases of white slough. The yellow slough was significantly thicker (4.0 vs. 0.8 mm, *p *< 0.01).

The thickness of the adhesions was examined using surgical materials for yellow and white slough. Nine cases of yellow slough and 20 cases of white slough were analyzed using surgical specimens. Of the nine cases of the yellow slough, seven were dMMR/MSl‐high and BRAF mt CRCs, and two were pMMR/MSS/MSI‐low and BRAF wt CRCs. In contrast, in 20 cases of white slough, one case was d‐MMR/MSl‐high and BRAF mt CRC, two cases were dMMR/MSI‐high BRAF wt CRCs, and 17 cases were pMMR/MSS/MSI‐low and BRAF wt CRC cases. The mean (±2 standard error) thickness of the adherent material was 4.0 mm (±1.8 mm) for yellow slough and 0.8 mm (±0.5 mm) for white slough, suggesting that cases of yellow slough were significantly thicker (*p *< 0.01; Figure 7).

## DISCUSSION

This study was performed to classify CRC into subtypes according to MMR/MSI states, RAS mutations, and BRAF mutations and clarify the differences in the endoscopic characteristics of each subtype, especially dMMR/MSI‐H and BRAF mt CRCs. In this study, dMMR/MSI‐H and RAS wt/BRAF mt CRC tended to be prominent in older adults, females, and in the right‐sided colon, consistent with previous findings (Table 1). There was no significant bias in the type of scope used for yellow slough cases and white slough cases that underwent endoscopic examination at our hospital (*p *= 0.238).

Furthermore, significantly more dMMR/MSI‐H and BRAFmt CRC cases showed endoscopic yellow slough than negative cases of both dMMR/MSI‐H and BRAFmt (*p *< 0.001), suggesting that yellow slough is an endoscopic feature of dMMR/MSI‐H and BRAFmt CRCs. More dMMR/MSI‐high positive CRC cases showed yellow slough even if BRAF mt was negative, compared with CRC cases that were negative for both dMMR/MSI‐H and BRAF mt (Figure 3). In the multivariate analysis, dMMR/MSI‐H positivity and BRAF mutation were independent risk factors for yellow slough (Figure 4). In this analysis, Firth regression was used due to the small sample size (*n* = 15). Power analysis (odds ratio = 30) showed > 99.9% power, indicating sufficient sensitivity.

Nevertheless, it should be noted that the small sample size limited the statistical power of the multivariate analysis, resulting in wide confidence intervals and reduced reliability of the risk estimates.

Furthermore, histologically, the slough was significantly thicker in patients with yellow slough than in those with white slough, and coagulative necrosis was observed only in those with yellow slough. This suggests that the tumor has a high proliferative potential, and coagulative necrosis was observed in many cases showing yellow granuloma. This finding is also consistent with the fact that has been reported that tumor mutation burden in the BRAF‐positive group was significantly higher than that in the BRAF‐negative group in dMMR/MSI‐high CRC cases.^18^

Mucus is attached to the bottom of the stomach and duodenal ulcers; are also observed in ulcer surfaces of the stomach, large intestine, and malignant tumors. Necrosis is broadly classified into coagulative, melting, caseous, fatty, fibrous, and gangrenous necroses. Slough is commonly observed at ulcer bases in the gastrointestinal tract, including malignant tumors. Among various types of necrosis, coagulative necrosis is characterized histologically by preserved cell outlines, eosinophilic cytoplasm, and nuclear changes such as condensation and disappearance.^19^ Granular necrosis, a form of coagulative necrosis seen in prolonged ischemia‐like myocardial infarction,^20^ appears microscopically within 4–6 hours. It is characterized by the presence of eosinophilic cytoplasm, loss of nuclei, and preserved tissue structure.^21^ In this study, histology showed these features, indicating tumor coagulative necrosis. (Figure 5). According to Askanazy,^22^ Boyd,^23^ and Okabayashi,^24^ the bed of a benign ulcer is divided into four layers: the first being the exudative layer; the second, third, and fourth layers are the fibrinoid necrosis, granulation, and scar layers, respectively. Askanazy^21^ stated that the first layer comprises red blood cells, white blood cells, and fibrin. The structure of the first and second layers, which are close to the surface, can determine the endoscopic characteristics, particularly the color tone. However, Lucas et al. reported that in upper gastrointestinal endoscopy, when the color tone of the ulcer base is uniform and white, there are no red blood cells in the first layer; when it is black or brownish, there are a large number of red blood cells in the first layer.^25^ Although there are a few reports of the ulcer base material in malignant tumors, the superficial structure of the first exudative layer and the second fibrin necrotic layer are present in the same form as benign ulcers, and it is thought that the color is determined by the surface layer. In this study, in cases that presented with yellow slough, there were many that histologically presented with coagulative necrosis of the tumor in the superficial structure of the ulcer, suggesting a relationship with yellow slough. dMMR/MSI‐H and BRAF mt CRC are lesions in the serrated pathway that develop from sessile serrated lesions (SSLs) into early‐stage lesions.^12^ SSLs show lateral expansion with a flat morphology on endoscopy and often have yellowish mucus adhering to them. Yellowish mucosal adherents result from the mucus produced by SSLs and are influenced by fecal substances. Mucus production is likely retained in the counterparts of advanced SSL, dMMR/MSI‐high, and BRAF mt CRC, and mucus adhesion in the exudative layer of the ulcer may also modify the yellowish characteristic.

In the NICHE2 study on dMMR/MSI‐H CRC, the 3‐year disease‐free survival rate was 100% in patients with dMMR CRC without distant metastasis treated with nivolumab + ipilimumab.^6^ In the CheckMate 8HW trial, the combination of nivolumab and ipilimumab was found to be associated with longer progression‐free survival than chemotherapy or nivolumab monotherapy in patients with MSI‐H or dMMR metastatic CRC who had not received prior systemic therapy.^5^, ^26^ The presence or absence of dMMR/MSI‐H is a factor influencing the treatment strategy for CRCs; therefore, it is extremely important for clinicians to understand the endoscopic characteristics. At present, the CRC treatment guidelines recommend testing for RASRAFMMR/MSI (HER2) in cases of advanced recurrent colorectal cancer that is not amenable to treatment. Nevertheless, the timing of these tests varies from one facility to another and from physician to another. If the likelihood of dMMR/MSI‐H could be predicted at the initial endoscopic examination, it would substantially increase the opportunity to perform molecular testing at the time of biopsy.

## CONCLUSION

No published reports exist on the endoscopic features of advanced CRCs that are dMMR/MSI‐H and BRAF mutation‐positive, particularly in relation to the presence of yellow slough. Although the number of cases was small, the observed differences were significant. Yellow slough may indicate dMMR/MSI‐H and BRAF mutations, suggesting the need for molecular testing.

## CONFLICT OF INTEREST STATEMENT

None.

## ETHICS STATEMENT

This study was conducted in accordance with the guidelines of the Declaration of Helsinki and approved by the Ethics Review Board of Fukuyama Medical Center (ERB2024027).

## PATIENT CONSENT STATEMENT

N/A

## CLINICAL TRIAL REGISTRATION

N/A
